# Differential Protective Effects of Edaravone in Cerebellar and Hippocampal Ischemic Injury Models

**DOI:** 10.1007/s12311-025-01804-3

**Published:** 2025-02-18

**Authors:** Jens Dickmeiß, Yoshiyuki Henning, Sarah Stahlke, Thomas Weber, Carsten Theiss, Veronika Matschke

**Affiliations:** 1 Department of Cytology, Institute of Anatomy, Medical Faculty, Ruhr-University Bochum, Bochum, Germany; 2 Institute of Physiology, University Hospital Essen, University of Duisburg-Essen, Essen, Germany; 3 Department of Anesthesiology and Intensive Care Medicine, Ruhr University Bochum, St. Josef Hospital, D-44791 Bochum, Germany; 4 International Graduate School of Neuroscience (IGSN), Ruhr-University Bochum, 44801 Bochum, Germany

**Keywords:** Brain damage, Stroke, Cell death, Hypoxia chamber, Ischemia, Organotypic slice cultures, Oxygen consumption rate (OCR), Seahorse XF, Mitochondria

## Abstract

Ischemic stroke is a leading cause of mortality and disability, with cerebellar strokes posing severe complications such as herniation and brainstem compression. Edaravone, a radical scavenger known for reducing oxidative stress, has shown neuroprotective effects in cerebral strokes, but its impact on cerebellar strokes remains unclear. This study investigates Edaravone’s protective properties in organotypic slice cultures of rat cerebellum and hippocampus, employing an oxygen-glucose deprivation (OGD) model to simulate ischemic stroke. The hippocampus served as comparative structure due to its high hypoxia sensitivity. Our results confirmed effective hypoxic induction with increases in HIF-1α and HIF-2α expression. Edaravone significantly reduced lactate dehydrogenase (LDH) levels, indicating diminished cellular damage, with cerebellar tissues showing greater vulnerability. Additionally, Edaravone reduced reactive oxygen species (ROS) in both tissues, though its efficacy may be limited by higher oxidative stress in cerebellar cultures. Seahorse XF analysis revealed that Edaravone preserved mitochondrial respiration and tissue integrity in cerebellar and hippocampal slice cultures. However, Edaravone was more effective in preserving mitochondrial respiration in hippocampal slices, suggesting that OGD-induced damage is more severe in cerebellar tissue. In conclusion, Edaravone demonstrates significant cell protective effects in both cerebellar and hippocampal tissues under OGD conditions, preserving tissue integrity and enhancing mitochondrial function in a tissue-dependent manner. These findings suggest Edaravone as a promising therapeutic candidate for cerebellar stroke. Further in vivo studies are required to assess its full clinical potential.

**Supplementary Information:**

The online version contains supplementary material available at 10.1007/s12311-025-01804-3.

## Introduction

Stroke is one of the leading causes of death worldwide and the primary cause of acquired and permanent disability in adults. According to the World Health Organization (WHO), a stroke occurs every two seconds worldwide, and someone dies from a stroke every six seconds, surpassing the combined mortality rates of malaria, tuberculosis, and HIV/AIDS [1]. Survivors often face significant limitations in their ability to live independently, burdening healthcare systems with extensive medical and nursing needs. Ischemic strokes, caused by intravascular thrombosis or embolism blocking a cerebral vessel, account for over 80% of strokes [2]. The clinical presentation and symptoms of a stroke largely depend on the affected brain region. Cerebellar ischemic stroke involving one or more of the three main cerebellar arteries (posterior inferior cerebellar artery (PICA), anterior inferior cerebellar artery (AICA) and/or superior cerebellar artery (SCA)) [3], are relatively rare, accounting for 2–3% of strokes, but they cause disproportionately high morbidity and mortality [4, 5]. The distinct symptoms of cerebellar strokes, such as dizziness, nausea or vomiting are often mistaken for less severe conditions, complicating diagnosis [3]. Treatment options for cerebellar strokes generally mirror those for cerebral strokes, including lysis therapy and mechanical thrombectomy. However, cerebellar strokes pose a higher risk of complications such as herniation and brain stem compression, requiring specialized therapeutic approaches. Cerebral oedema resulting from strokes can lead to ventricular system compression, requiring additional therapeutic measures [4–6]. The pathophysiology of hypoxic neuronal brain damage in ischemic strokes involves several signaling pathways leading to necrosis and apoptosis, including energy deficiency, loss of ion homeostasis, acidosis, increased intracellular calcium levels, excitotoxicity, free oxygen radicals, complement activation, cytokine-mediated mechanisms, and immune system activation. These complex mechanisms can be categorized into three basic mechanisms: direct neuronal loss through apoptotic signaling pathways [7], production of reactive oxygen species (ROS) causing oxidative stress, and ischemia-induced inflammation of neuronal tissue [8]. Massive cell damage occurs not only during acute ischemia but also during reperfusion, exacerbated by ROS and neuroinflammatory processes [9]. Expanding current therapies to target these pathophysiological processes could significantly advance stroke treatment. Edaravone (3-methyl-1-phenyl-pyrazolin-5-one) represents a promising option for innovative stroke therapy. Approved by the US *Food and Drug Administration (FDA)* in 2017 for treatment of amyotrophic lateral sclerosis, Edaravone acts as a radical scavenger, mitigating oxidative stress [10]. Its favorable acid dissociation constant (pKa) allows it to exist in both lipophilic and hydrophilic forms, facilitating passage through cell membranes and the blood-brain barrier [11]. Edaravone significantly reduces oxidative stress during a stroke, lowering cell apoptosis and tissue damage. Further, it inhibits vascular endothelial growth factor (VGEF) expression in astrocytes, protecting against brain oedema [12]. It also increases the expression of BDNF and Bcl-2, which have anti-apoptotic effects, and reduces caspase-3 activity, crucial for apoptosis. Edaravone likely lowers post-ischemic leukotriene levels, reducing oedema, with 5-LOX activation playing a key role in regulating arachidonic acid metabolism [13]. Although Edaravone has shown promise in human trials for cerebral strokes, its potential in cerebellar strokes remains underexplored, presenting an opportunity to advance stroke treatment [14–17]. This research aims to investigate Edaravone’s protective effects in cerebellar ischemic stroke using a hypoxia model with organotypic slice cultures. The hypoxia-sensitive hippocampus will also be used as a comparative structure [18] to further clarify its treatment efficacy.

## Materials and Methods

### Animals

All procedures were conducted under established standards of the German federal state of North Rhine Westphalia, in accordance with the European Communities Council Directive 2010/63/EU on the protection of animals used for scientific purposes. According to current German and European legislation, the removal of organs or cells from vertebrates for scientific purposes is not considered an animal experiment if the animals have not been subject to surgical interventions or invasive treatments prior to sacrifice. Thus, the euthanasia of rats for the removal of brain tissue does not require approval or permission from local or federal authorities.

Wistar rats of both sexes from our in-house breeding stock were used and housed under a 12:12 light-dark cycle with food and water provided ad libitum. For organotypic slice culture preparation, rats aged postnatal days 7–10 (P7–10) were utilized. In accordance with established ethical standards and relevant German and European legislation, the animals were decapitated without prior euthanasia.

### Organotypic Cell Cultures and Oxygen-Glucose Deprivation (OGD)

For this study, organotypic slice cultures of the cerebellum and hippocampus were prepared from seven Wistar rats following the method described by Wolters and Reuther et al. [19]. In some preparations, an additional animal was used to ensure consistent slice quality. Brain tissue was sectioned using a McLain Tissue Chopper to obtain cerebellar slices (275 μm thickness) and hippocampal slices (350 μm thickness), which were subsequently cultivated on inserts (Millicell; REF: PICM0RG50) in six-well plates containing 1 ml of culture medium per well. The culture medium consisted of 25% heat-inactivated horse serum, 25% Hanks’ Balanced Salt Solution (Sigma; REF: H8264), 2.6% glucose (Merck; REF: 8342), 1% Nerve Growth Factor, 1% penicillin, 1% glutamine (Gibco; REF: 35050-061), 0.2% phenol red (Sigma; REF: P0290) in Basal Medium Eagle (Sigma; REF: B1522). Cultures were maintained in an incubator at 5% CO_2_ and 37 °C for seven days. To ensure robust and unbiased experimental conditions, slices were randomly pooled and distributed across wells. Importantly, each well contained either four cerebellar or five hippocampal slices, with slices in a single well always originating from different rats to prevent intra-animal redundancy. This distribution strategy ensured that slices from different animals were evenly represented across all experimental groups. Only slices with a preserved architecture were included, enhancing the reproducibility and reliability of the experimental results. A minimum of three wells per condition was prepared for each experimental group, and each experiment was independently repeated at least three times with new preparations, accounting for biological variability across animals and technical variability across experimental runs. After seven days in culture, oxygen-glucose deprivation (OGD) was induced using two specific media formulations. The OGD medium lacked glucose and contained 25% Hanks’ Balanced Salu Solution (Sigma; REF: H8264], 1% Nerve Growth Factor, 1% penicillin, 1% glutamine (gibco; REF: 35050-061), 0.2% phenol red (Sigma; REF: P0290) in Dulbecco’s Modified Eagle’s Medium (Sigma; REF: D6046). A control medium with identical composition but with glucose supplementation (6.5 mg/ml) was used for normoxic conditions. Both media were pre-gassed for at least one hour before use. The OGD procedure was conducted in a hypoxic chamber (Hypoxic Chamber Polymer O_2_ Control Glove Box; Coy Lab, Grass Lake, MI, USA) under controlled conditions of 37 °C, 5% CO_2_ and 0.4% O_2_. Following the designated OGD duration, the inserts were transferred back to normoxic conditions with fresh glucose-containing medium to initiate reoxygenation. Experimental time points and protocols were conducted as specified in the respective test procedures.

### Real Time Quantitative PCR

To evaluate the impact of OGD on the expression of hypoxia-inducible factors (HIF), qPCR was performed. Total RNA was extracted from organotypic slice cultures of the cerebellum and hippocampus after 1, 4, and 8 h of OGD, as well as from normoxic control conditions. Each well representing one timepoint included 4 cerebellar and 5 hippocampal slice cultures. The isolated RNA was further processed to analyse the mRNA levels of HIF-1α and HIF-2α using the RNA isolation kit from Machery-Nagel (REF: 740955.50). The manufacturer’s protocol was followed with slight modifications, particularly an adjusted elution volume of 40 µl to achieve a higher RNA concentration. cDNA was synthesized using oligo(dT) primers and the GoScript Reverse Transcription Mix (Promega: A2790). qPCR was performed using the GoTaq qPCR Master Mix (Promega: REF A6002). Primer sequences were as follows: *Hif1a*: forward 5’-AAT GTA CCC TAA CAA GCC GGG–3’, reverse 5’-GTT TCT TGT AGC CAC ACT GCG-3’; *Hif2a*: forward 5’- AGT GGT CTG TGG GCA ATC AG-3’, reverse 5’-AAC ATG GAG ACA TGA GGC GG-3; *Actb*: forward 5’-CTA AGG CCA ACC GTG AAA AG-3’, reverse 5’-AAC ACA GCC TGG ATG GCT AC-3’. The differences in the expression levels were calculated using the 2^-ΔΔCt^ method with endogenous normalization to Actin.

### Western Blotting

To prevent the degradation of HIF-1α and HIF-2α proteins during the transition to normoxic conditions, protein isolation was performed directly within the hypoxia chamber. Organotypic slice cultures were carefully removed from the cultivation membranes by pipetting with PBS containing a protease and metalloprotease inhibitor cocktail (ThermoFisher; Halt Protease Inhibitor Cocktail (100X); REF: 87786). The detached samples were transferred to Eppendorf tubes, and excess PBS was removed. The samples were then snap-frozen in liquid nitrogen. Each sample consisted of either 5 hippocampal or 4 cerebellar organotypic slice cultures.

Frozen slices from the rat cerebellum and hippocampus were homogenized in 100 µL of lysis buffer with a protease/phosphatase inhibitor cocktail (Cell Signaling Technology, REF: 5872 S). The lysates were incubated on ice for 2 h in a rotating incubator, followed by centrifugation at 13,400 × g for 20 min at 4 °C. Supernatants were stored at -80 °C until further use. For SDS-PAGE, 30 µg of total protein were incubated in Laemmli sample buffer (0.1 M Tris, pH 6.8, 4% SDS, 10% β-mercapto-ethanol, 0.025% bromophenol blue, 20% glycerol, 5% ddH2O) for 5 min at 95 °C. Proteins were separated by SDS-PAGE and transferred onto a PVDF membrane using a Trans-Blot Turbo Transfer System (Bio-Rad Laboratories). Membranes were blocked with 5% skim milk in TBS-T for 1 h at room temperature. Primary antibodies against HIF-1α (Cayman Chemical, REF: CAY-10006421), HIF-2α (Novus Biologicals, REF: NB100-122), and Actin (Sigma-Aldrich, REF: A2103) were diluted in blocking buffer and incubated overnight at 4 °C. Secondary antibodies—goat anti-mouse (Sigma-Aldrich, REF: A2304) and goat anti-rabbit (Sigma-Aldrich; REF: A0545)—were diluted in blocking buffer and incubated for 1 h at room temperature. Protein signals were developed using SuperSignal West Femto Maximum Sensitivity Substrate (Thermo Fisher Scientific; REF: 34096) and detected with the Fusion FX System (Vilber). Band intensities were measured using ImageJ2 (version 2.9.0, National Institutes of Health, Bethesda), with normalization to actin blots as a loading control. Full, uncropped Western Blots for HIF-1α, HIF-2α, and the corresponding Actin bands are shown in Supplementary Fig. 1.

### LDH Assay

The organotypic slice cultures were prepared as previously described, and before the oxygen-glucose deprivation (OGD) procedure, the samples were divided into two groups: an OGD group and a control group. Each group contained four inserts—two inserts with 4 cerebellar slice cultures and two with 5 hippocampal slice cultures each. Before transferring the OGD group inserts to the hypoxia chamber, 2 µl of media were collected from all wells to determine the background signal. The samples were stored in 48 µl of storage buffer at -80 °C. The storage buffer consisted of Tris-HCL (200mM, pH 7.3) (Fisher BioReagents; REF: BP153-1), 10% glycerol (J.T.Baker) & 1% BSA (ROTH; REF: 8076.2).

**Fig. 1:**
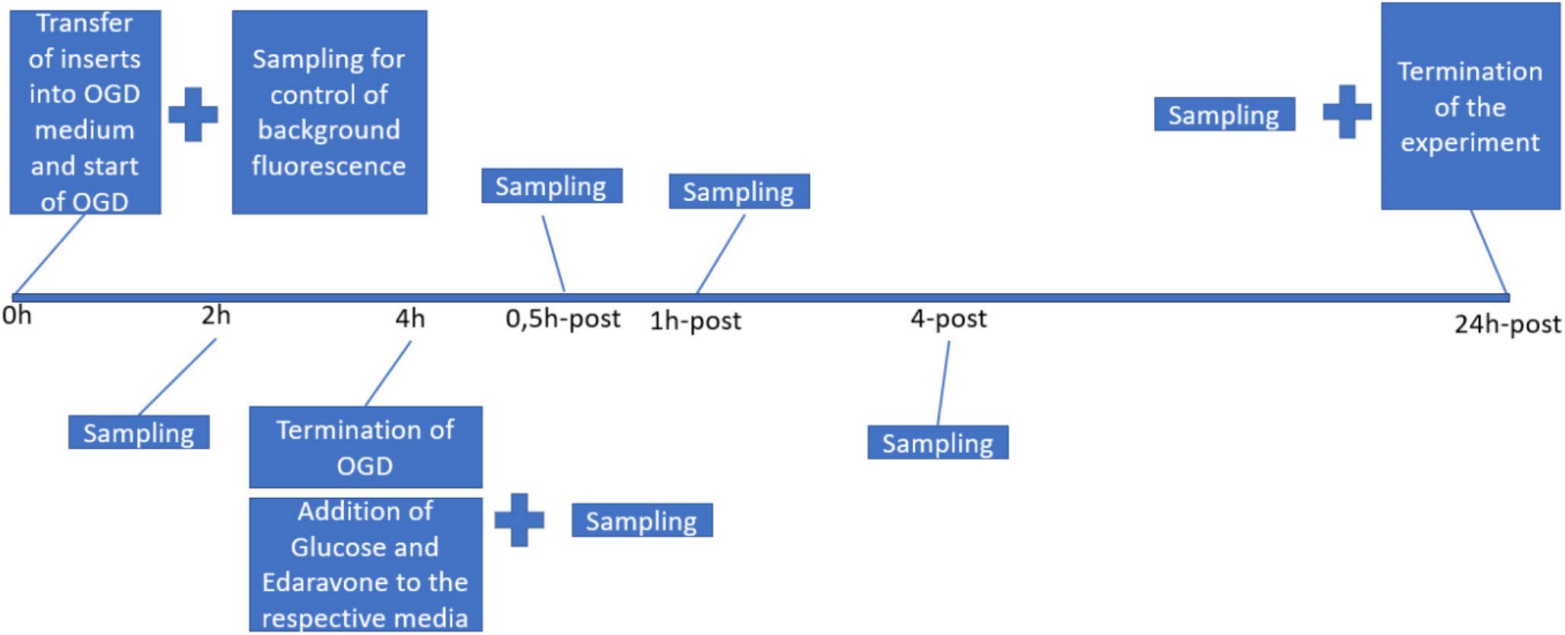
Experimental timeline for an Oxygen-Glucose Deprivation (OGD) study. The sequence starts with the transfer of inserts into the OGD medium and the initiation of OGD, followed by sample collection at different time points (0, 2, and 4 h). After the termination of OGD at 4 h, glucose and Edaravone are added to the respective media. Additional samples are collected at various time points post-OGD (0.5, 1, 4, and 24 h). The experiment concludes with the final sample collection at 24 h post-OGD

The control group remained under normoxic conditions in the previously described control medium. The OGD group was placed in the hypoxia chamber for 4 h using the established OGD medium. During this period, samples were taken after 2 h and 4 h under hypoxic conditions. After 4 h, the six-well plates were removed from the hypoxia chamber, and 26 µl of glucose (250 mg/ml) were added to the wells to simulate reoxygenation. Additionally, 1 µl of Edaravone (10 mM) (Aldrich; REF: M70800-5G) was added to the intervention parts of both the control and OGD groups, resulting in a final concentration of 10 µM Edaravone. This setup created an intervention group in both the OGD and control conditions, while the remaining OGD and control groups did not receive Edaravone. As illustrated in Fig. 1 further samples were taken 30 min, 1 h, 4 h, and 24 h after reoxygenation, and stored in 48 µl of storage buffer at -80 °C. The samples were then analyzed using the LDH-Glo Cytotoxicity Assay (Promega; REF: J2380), according to the manufacturer’s instructions. In the assay we used the recommended volumes of 50 µl LDH detection reagent and 50 µl sample with a 60 min incubation time.

Raw fluorescence values of each experimental group were normalized relative to the control group, with the mean values of the control group defined as 100%. This normalization allows a comparable representation of the results across different conditions.

### ROS Assay

The organotypic slice cultures were subjected to OGD as previously described. After 4 h of OGD, the samples were reoxygenated by placing them back in the normoxic incubator and adding 26 µl of glucose (250 mg/ml) to the culture medium. At the same time, the intervention groups received 1 µl of Edaravone (10 mM) (Aldrich; REF: M70800-5G). Following reoxygenation, the samples were incubated for 4 h under these conditions, after which they were snap-frozen in liquid nitrogen. The samples were treated with 200 µl PBS and 1 µl of 1% Triton X-100, followed by sonication on ice. Insoluble particles were removed after centrifugation at 10,000 × g for 5 min. Subsequently, the samples were analyzed using the ROS assay OxiSelect In Vitro ROS/RNS Assay Kit (Green Fluorescence; REF: STA-347-T) according to the manufacturer’s instructions with a 45 min incubation time. The raw fluorescence values of each experimental group were normalized relative to the control group, with the mean values of the control group defined as 100%. This normalization allows for a comparable representation of the results across different conditions.

### Seahorse Metabolic Flux Analysis of Organotypic Slice Cultures

To assess mitochondrial respiration, oxygen consumption rate (OCR) was measured in cerebellar and hippocampal organotypic slice cultures using the Seahorse XFe24 Analyzer (Agilent Technologies, USA). After the OGD procedure, slices were subjected to a 4-hour reperfusion phase in Seahorse XF DMEM medium (Agilent; REF: 103575-100) containing 36 mM glucose and 1x GlutaMAX (Thermo Fisher Scientific; REF: 35050061), with or without 10 µM Edaravone. Following the reperfusion period, culture inserts were quickly washed in Assay Medium (Seahorse XF DMEM medium, 5 mM glucose, 1x GlutaMAX) prior to collecting 1 mm tissue punches using a biopsy punch. Tissue punches were transferred to Seahorse XF24 Islet Capture Microplates (Agilent; REF: 101122-100) in 500 µL Assay Medium and secured using specialized mesh inserts to ensure consistent positioning within the wells during measurement. Following an 1 h equilibration period in a cell culture incubator (21% O_2_, 0% CO_2_, 37 °C), OCR was measured in response to sequential injections of metabolic modulators, including glucose (36 mM), pyruvate (10 mM), rotenone/antimycin A (Rot/AA; inhibitors of mitochondrial complexes I and III; 2 µM each), and 2-deoxy-D-glucose (2-DG; a glycolysis inhibitor; 100 mM). Measurements (3x baseline, 5x after each injection) consisted of the following phases: Mix 2 min, Wait 2 min, Measure 5 min. Each condition was measured three times using tissue punches derived from three different animals to ensure biological replicates and statistical reliability. Raw OCR values were normalized to DNA content of the respective tissue punch to account for potential differences in tissue integrity and cell number. Basal respiration was calculated by subtracting the last OCR value after Rot/AA injection from the last baseline OCR level. Glucose-induced respiration was calculated by subtracting the difference between the last baseline OCR value and the highest glucose-induced OCR value and pyruvate-induced OCR was calculated by subtracting the last glucose-induced OCR from the highest pyruvate-induced OCR value.

### Statistical Analysis

The data were analyzed statistically in accordance with the methodology previously described [20]. Data analysis was conducted using GraphPad Prism 9 software (GraphPad Software, USA). The results are presented as mean values from at least three independent experiments, along with the standard error of the mean (SEM). Kolmogorov-Smirnov normality test was used to confirm normal distribution. To assess statistical significance between two groups, a two-tailed Student’s t-test was applied, while comparisons across multiple groups were performed using a one-way analysis of variance (ANOVA). Pairwise comparisons were conducted using Tukey’s post-hoc test. A p-value of less than 0.05 was considered indicative of statistical significance.

## Results

### OGD Effectively Induces Expression of Hypoxia Inducible Factors in Cerebellar and Hippocampal Organotypic Slice Cultures

To confirm that the OGD procedure effectively induces hypoxia and oxygen deprivation in the tissue, the expression levels of HIF-1α and HIF-2α were assessed at both the mRNA level, using qPCR, and the protein level, using Western blot analysis. mRNA expression was measured after 1, 4, and 8 h of OGD to determine the optimal duration of OGD that induces a consistent hypoxic response in the organotypic slice cultures for subsequent experiments.

**Fig. 2:**
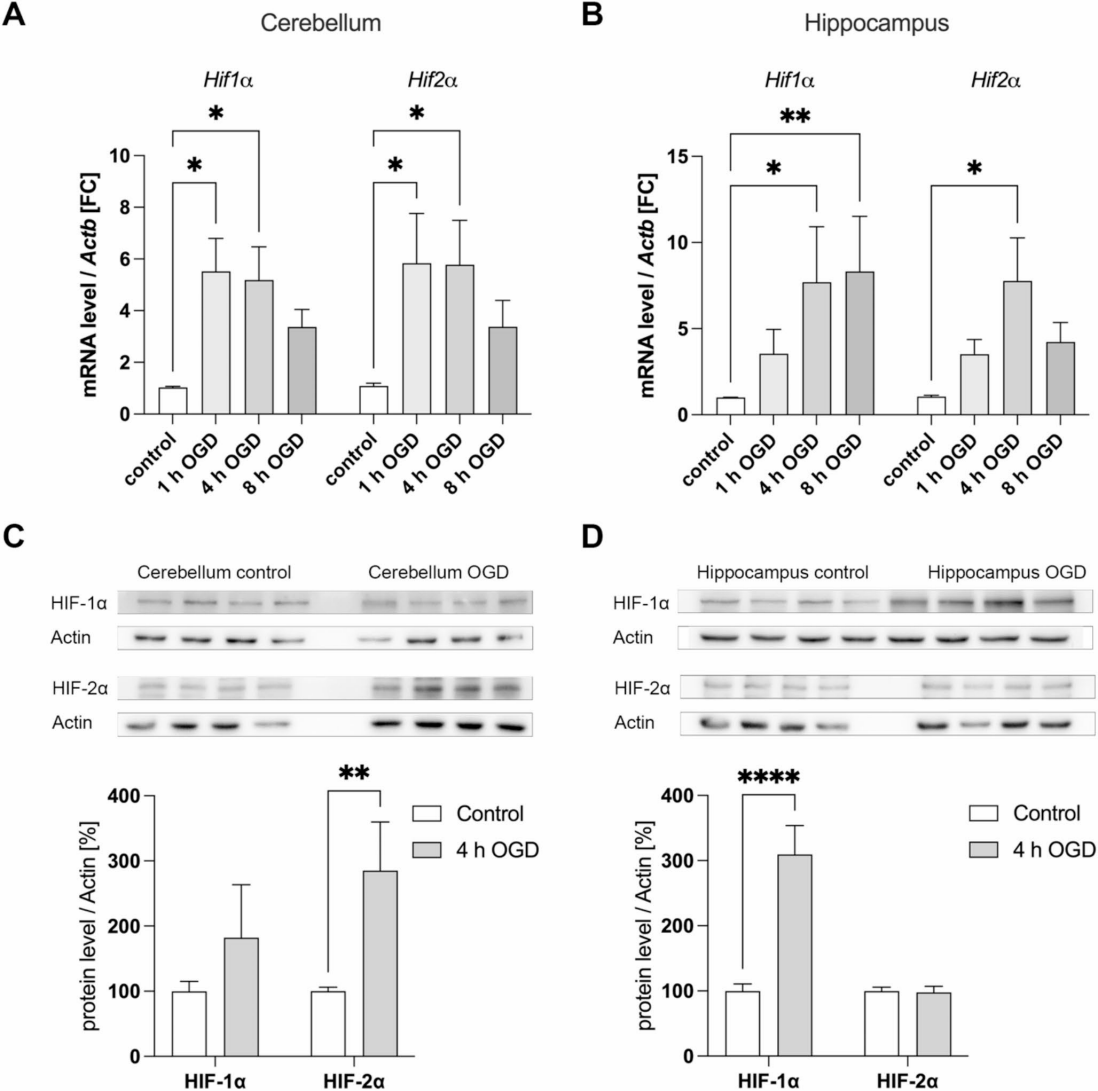
HIF-1α and HIF-2α expression levels showed a tissue-specific upregulation following OGD in cerebellar and hippocampal organotypic slice cultures. (**A**) *Hif1a* and *Hif2a* expression levels normalized to *Actb* in cerebellar samples after 1, 4, and 8 h of OGD. Significant upregulation of *Hif1a* and *Hif2a* was observed at 1 h and 4 h of OGD. (**B**) *Hif1a* and *Hif2a* expression levels normalized to *Actb* in hippocampal samples after 1, 4, and 8 h of OGD. *Hif1a* showed significant upregulation at 4 h and 8 h of OGD, while *HIF2A* was significantly upregulated only at 4 h of OGD. (**C**) Representative Western blot images for HIF-1α and HIF-2α in cerebellar slice cultures after 4 h of OGD. Semiquantitative analyses indicated a trend toward increased HIF-1α protein levels and a significant upregulation of HIF-2α. Actin was used as loading control. (**D**) Representative Western blot images of HIF-1α and HIF-1α in hippocampal slice cultures after 4 h of OGD. Semiquantitative analyses showed a significant increase in HIF-1α protein levels, with no significant changes in HIF-2α protein expression. Actin was again used as a loading control. Data are expressed as mean ± SEM; *p < 0.05, **p < 0.01, ****p < 0.0001 (**A**, **B** One-way ANOVA with Tukey’s multiple comparisons test; **C**, **D** two-tailed Student’s t-test). (**A**, **B**) *n* = 40 cerebellar and 35 hippocampal slice cultures per group, five independent preparations; (**C**, **D**) *n* = 16 cerebellar and 20 hippocampal slice cultures per group, four independent preparations

qPCR analysis of the cerebellar organotypic slice cultures revealed a significant increase in HIF-1α and HIF-2α mRNA levels in the samples subjected to OGD. This upregulation of HIF expression was detectable as early as 1 h of OGD and persisted through 4 and 8 h. However, a decrease in *Hif1a* and *Hif2a* expression levels was observed after 8 h (Fig. 2A). In hippocampal slice cultures, qPCR similarly showed a significant increase in *Hif1a* and *Hif2a* levels following OGD. Unlike the cerebellar samples, both *Hif1a* and *Hif2a* expression continued to increase from 1 to 4 h of OGD in the hippocampus. *Hif1a* levels continued to rise after 8 h of OGD, whereas *HIF2A* expression decreased between 4 and 8 h (Fig. 2B). Since significant mRNA upregulation was observed at the 4 h time point in both cerebellum and hippocampus, this duration was selected for subsequent experiments.

Western blot analysis confirmed the findings from qPCR. In the cerebellar slice cultures, HIF-1α protein levels increased after OGD, though this increase did not reach statistical significance, while HIF-2α protein expression was significantly elevated after 4 h of OGD (Fig. 2C). In hippocampal slice cultures, Western blots showed a significant increase in HIF-1α protein levels in the OGD-treated samples. In contrast, no significant changes were observed in HIF-2α protein expression in the hippocampus (Fig. 2D).

### Edaravone Displays Protective Properties in both Cerebellar and Hippocampal Organotypic Slice Cultures after OGD

To investigate whether Edaravone exhibits protective properties on organotypic slice cultures of the cerebellum and hippocampus following OGD-reperfusion conditions, the drug was added to the culture medium after a 4 h OGD period. The protective effect was assessed using an LDH assay, allowing for a quantitative comparison of cell damage between the treatment and control groups.

**Fig. 3:**
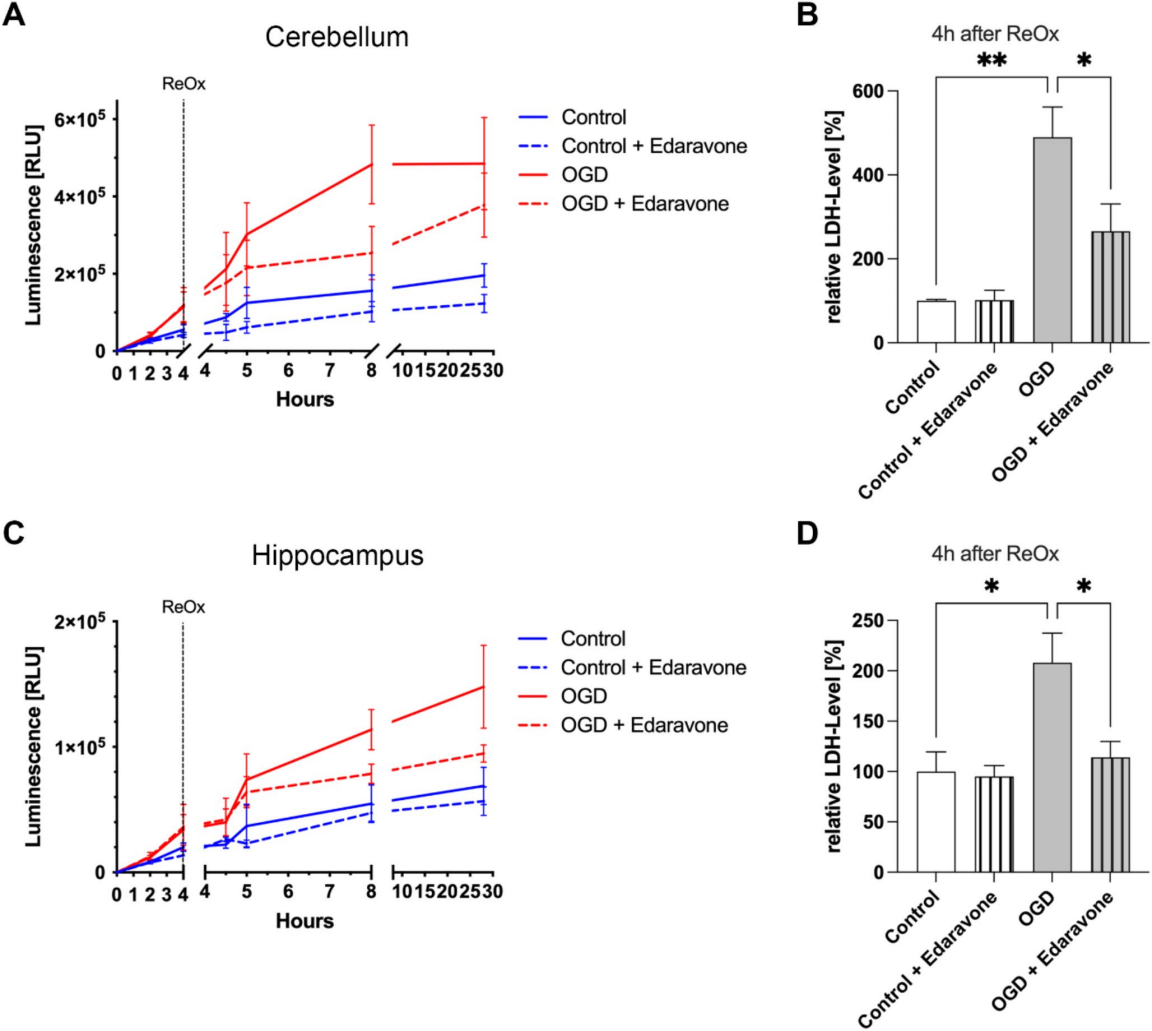
Edaravone significantly reduced LDH levels and cell damage in organotypic slice cultures of the cerebellum and hippocampus following OGD. (**A**) Time course of LDH levels in cerebellar slices at different time points, with the X-axis representing time (h) and the Y-axis representing absolute luminescence values, indicating LDH levels. Over time, LDH levels increased in both the OGD and control groups, with Edaravone-treated samples consistently showing lower LDH levels compared to untreated samples. (**B**) Bar chart showing the relative LDH levels in cerebellar slices 4 h after reoxygenation (ReOx). Since hypoxia-induced cell damage was clearly evident at this point, the 4-hour post-reoxygenation time point was selected for analysis. Absolute LDH values were normalized to the control group and presented as percentages. Cerebellar samples in the untreated OGD group exhibited significantly elevated LDH levels compared to controls. A significant reduction in LDH levels was observed following Edaravone treatment, indicating a marked decrease in cell damage in cerebellar cultures. (**C**) Time course of LDH levels in hippocampal slices, showing increased LDH levels in the OGD groups compared to controls. (**D**) Bar chart showing the relative LDH levels in hippocampal slices 4 h after reoxygenation. Since hypoxia-induced cell damage was clearly evident at this point, the 4-hour post-reoxygenation time point was selected for analysis. Absolute LDH values were normalized to the control group and presented as percentages. In hippocampal samples, untreated OGD groups showed significantly elevated LDH levels compared to controls. Edaravone treatment resulted in a significant reduction in LDH levels, highlighting a substantial decrease in cell damage also in hippocampal cultures. Data are expressed as mean ± SEM; *p < 0.05, **p < 0.01 (One-way ANOVA with Tukey’s multiple comparisons test). (**A**, **B**) *n* = 20 slice cultures per group, five independent preparations; (**C**, **D**) *n* = 25 slice cultures per group, five independent preparations. ReOx in (**A**) and (**C**) indicates the time point of reoxygenation and glucose supplementation, and for the Edaravone group, the addition of Edaravone as well

In cerebellar slice cultures, samples subjected to OGD exhibited significantly higher LDH levels in the medium compared to non-OGD controls (Fig. 3A). Cultures treated with Edaravone demonstrated a reduction in LDH levels compared to untreated OGD cultures, indicating a protective effect of the drug over the duration of the experiment (Fig. 3A). Four h after reoxygenation, the OGD group showed a marked increase in LDH levels relative to the control, but a substantial reduction was observed in Edaravone-treated cultures compared to the untreated OGD group, further supporting the protective effect of Edaravone (Fig. 3B).

Similarly, hippocampal slice cultures exposed to OGD showed higher LDH concentrations over time compared to control groups. Edaravone treatment of OGD-treated slices led to significantly reduced LDH levels compared to untreated OGD cultures (Fig. 3C). This trend continued 4 h after reoxygenation, with Edaravone-treated hippocampal slices showing markedly lower LDH levels than untreated OGD cultures (Fig. 3D). No significant reduction in LDH levels was observed in either cerebellar or hippocampal slices not exposed to OGD following Edaravone treatment.

Comparing the extent of LDH level increase between the cerebellum and hippocampus, OGD-exposed cerebellar slices exhibited a 390% higher mean LDH level compared to non-OGD controls (Fig. 3B), while hippocampal slices showed a smaller increase of 108% (Fig. 3D). In terms of LDH reduction following Edaravone treatment, cerebellar cultures displayed a 45.9% decrease in mean LDH levels, whereas hippocampal cultures demonstrated a greater reduction of 54.9%.

### Edaravone Effectively Reduces ROS Levels in Cerebellar and Hippocampal Organotypic Slice Cultures After OGD

**Fig. 4:**
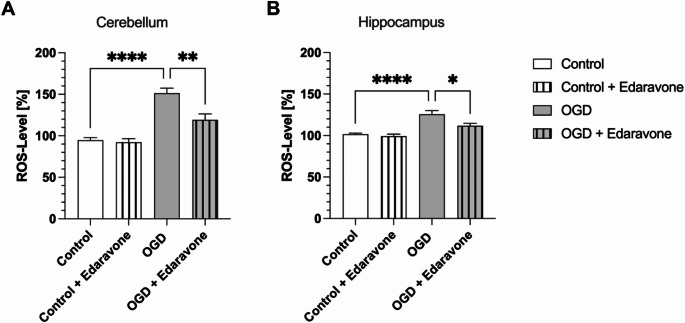
Edaravone significantly reduces ROS levels in both cerebellar and hippocampal organotypic slice cultures following OGD. (**A**) ROS levels in cerebellar slices, and (**B**) ROS levels in hippocampal slices, are shown for the control and OGD groups, with and without Edaravone treatment. ROS levels were normalized to the control group and set to 100%. As with the LDH assay, samples were collected 4 h post-reoxygenation, coinciding with 4 h after Edaravone application. In both tissues, OGD groups exhibited a significant increase in ROS levels compared to controls. The application of Edaravone in OGD groups led to a significant reduction in ROS levels in both cerebellum and hippocampus. Fluorescence values for ROS measurements were obtained in triplicates. Data are presented as mean ± SEM; *p < 0.05, **p < 0.01, ****p < 0.0001 (One-way ANOVA with Tukey’s multiple comparisons test). (**A**, **B**) *n* = 48 cerebellar and 60 hippocampal slice cultures per group, four independent preparations

To investigate the potential protective mechanism of Edaravone as a radical scavenger, ROS levels were analysed in the various treatment groups using a ROS assay. For comparison, ROS levels in the control groups were normalized to 100% for both cerebellar and hippocampal organotypic slice cultures. In the cerebellar cultures, a significant increase in ROS levels was observed in the OGD group compared to the control group (Fig. 4A). However, in the Edaravone-treated OGD group, ROS levels were significantly reduced compared to the untreated OGD group (Fig. 4A). A similar pattern was observed in hippocampal slice cultures (Fig. 4B). The OGD group exhibited a marked increase in ROS levels, while Edaravone treatment significantly reduced ROS levels compared to the untreated OGD group (Fig. 4B).

When comparing the magnitude of ROS level increase between the cerebellum and hippocampus in OGD-exposed samples without Edaravone, ROS levels in cerebellar slices were 39.1% higher than in non-OGD controls, while the increase in hippocampal slices was 25.8%. Edaravone treatment resulted in a 22.0% reduction in ROS levels in cerebellar slices, whereas the reduction in hippocampal slices was 11.2%.

### Tissue-Dependent Metabolic Response to Edaravone Treatment After OGD

To further elucidate the protective mechanisms of Edaravone, mitochondrial respiration was assessed using the Seahorse XFe24 Analyzer in cerebellar and hippocampal slice cultures subjected to OGD conditions, followed by reoxygenation with and without Edaravone treatment.

**Fig. 5:**
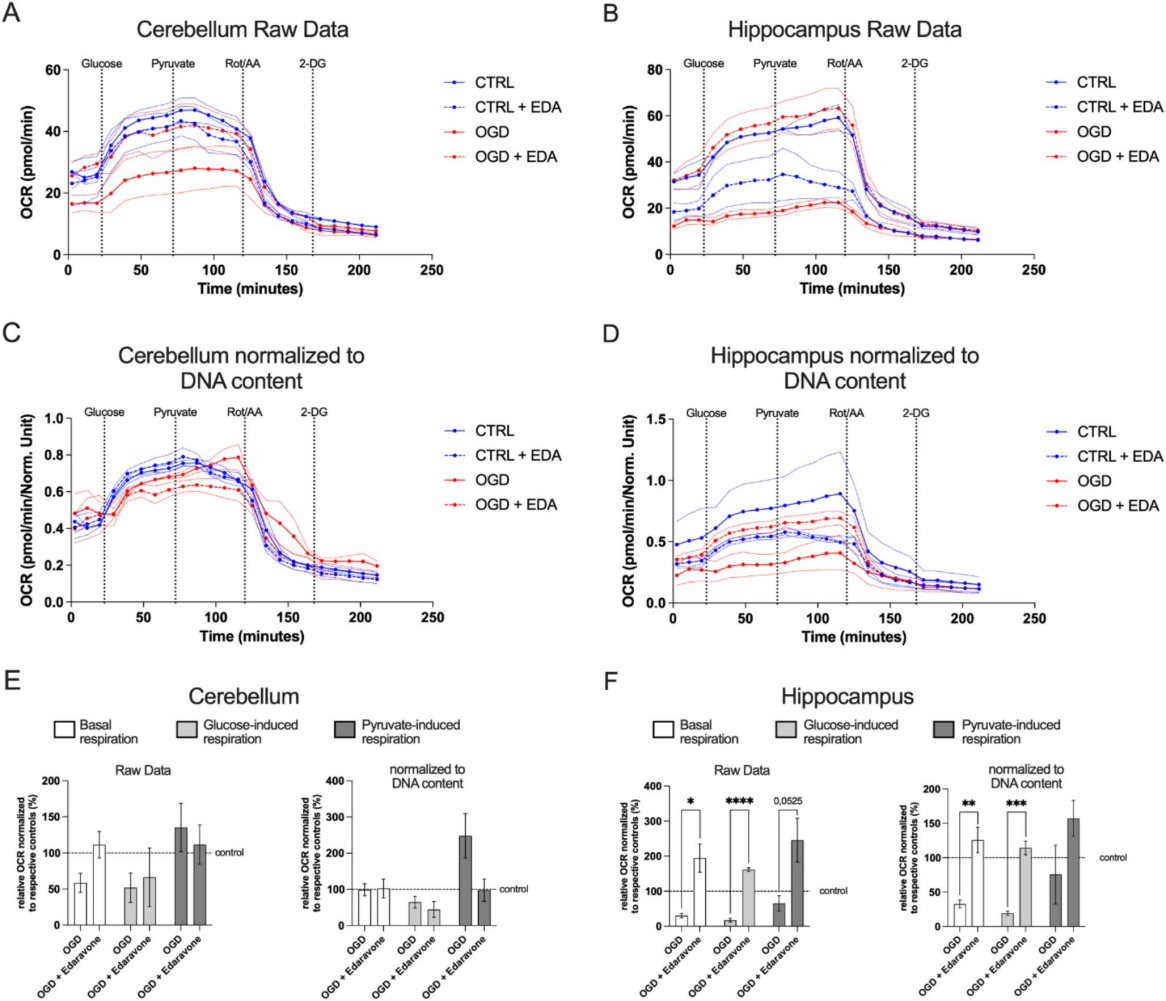
Tissue-Dependent Mitochondrial Response to Edaravone Treatment After OGD. (**A**, **B**) Raw oxygen consumption rate (OCR) data for cerebellar (A) and hippocampal (**B**) organotypic slice cultures under normoxic conditions (blue line), after OGD (red line), and with (dashed lines) or without (solid lines) Edaravone treatment. Thin dashed lines indicate the standard error of the mean (SEM). Time points of glucose, pyruvate, rotenone/antimycin A (Rot/AA), and 2-deoxy-D-glucose (2-DG) injections are indicated. (**C**, **D**) DNA-normalized OCR data for cerebellar (**C**) and hippocampal (**D**) cultures. In cerebellar slices, OGD led to a slight reduction in normalized OCR compared to normoxic controls. Edaravone treatment did not restore OCR, as OGD + Edaravone cultures exhibited similar respiration levels to the untreated OGD group. In hippocampal slices, OGD resulted in a decrease in OCR compared to normoxic controls, whereas Edaravone treatment restored OCR to control levels. (**E**) Basal and substrate-driven respiration in cerebellar slice cultures. Raw OCR (left) and normalized OCR (right) levels were expressed relative to their respective control groups. Edaravone treatment did not significantly improve respiration across all conditions. (**F**) Basal and substrate-driven respiration in hippocampal slice cultures. Raw OCR (left) and normalized OCR (right) levels were expressed relative to their respective control groups. Hippocampal slice cultures exhibited a significant increase in basal- and glucose-driven OCR following Edaravone treatment. Pyruvate-induced OCR showed a non-significant upward trend with Edaravone treatment. The 100% control values are indicated by a dashed line. Data are presented as mean ± SEM, *p < 0.05, **p < 0.01, **p < 0.001, ****p < 0.0001 (two-tailed Student’s t-test); *n* = 3 slice cultures per group

Following OGD, a marked decline in raw OCR was observed in both cerebellar and hippocampal slices (Fig. 5A, B). Edaravone treatment fully restored raw OCR levels in both tissues (Fig. 5A, B). To account for differences in cell density, OCR values were normalized to DNA content, reflecting mitochondrial respiration per cell rather than total tissue oxygen consumption. This normalization revealed distinct recovery patterns between the two tissues and helped differentiate whether Edaravone’s effects resulted from preserved cellular integrity or direct mitochondrial enhancement. In this DNA-normalized dataset, OCR in the OGD-group of cerebellar slice cultures (Fig. 5C) was reduced in the OGD group compared to normoxic controls, although not significant. Edaravone treatment during reperfusion did not restore OCR, as OGD + Edaravone cultures exhibited respiration levels similar to the untreated OGD group. This suggests that the observed reduction in raw OCR in cerebellar slices was primarily due to cell loss rather than direct mitochondrial dysfunction. In contrast, hippocampal slice cultures (Fig. 5D) showed a different response to Edaravone treatment. Following OGD, OCR was reduced compared to normoxic controls. In the DNA-normalized OCR levels, Edaravone-induced improvement of respiration was still apparent. This tissue-dependent metabolic response is further supported by the calculation of basal respiration, glucose-induced maximal respiration, and pyruvate-induced maximal respiration in OGD and OGD + Edaravone-treated groups. For this purpose, OCR levels of both groups were normalized to the respective control group to calculate relative change after treatment. Basal respiration analysis revealed distinct tissue-specific responses to Edaravone. In cerebellar slices (Fig. 5E), calculations based on raw OCR data showed that OGD led to a decrease in basal respiration compared to control data, though this reduction was not statistically significant compared to Edaravone-treated OGD slices. Still, Edaravone treatment restored basal respiration to control levels, consistent with its cell-protective effects. In the DNA-normalized dataset, basal respiration remained at approximately 100% across all experimental conditions, reinforcing that Edaravone’s primary effect in the cerebellum was the preservation of tissue integrity rather than direct mitochondrial enhancement. In contrast, hippocampal slices (Fig. 5F) responded differently to Edaravone treatment. In this tissue, Edaravone fully restored basal respiration of OGD slices and even increased it beyond control levels, resulting in significantly higher basal respiration compared to OGD slices without Edaravone treatment. This suggests that in the hippocampus, Edaravone supports mitochondrial function beyond cell protection, potentially through enhanced metabolic efficiency or mitochondrial biogenesis. Substrate-specific further underscored these tissue-dependent effects. In cerebellar slices (Fig. 5E), glucose-induced OCR was slightly reduced in both OGD groups, though the difference did not reach statistical significance, and pyruvate-induced maximal respiration remained unchanged across all groups. In hippocampal slices (Fig. 5F), Edaravone treatment significantly increased glucose-driven respiration following glucose injection in the OGD + Edaravone group compared to untreated OGD slice cultures. Pyruvate-induced respiration exhibited a trend toward higher values in the OGD + Edaravone condition, though this effect did not reach statistical significance. These findings suggest a tissue-specific response to Edaravone, with its primary effect in the cerebellum being the preservation of tissue integrity, whereas in the hippocampus, it appears to support mitochondrial respiration after ischemic stress.

## Discussion

Ischemic stroke is a leading cause of disability and death worldwide, with the cerebellum being particularly vulnerable due to its critical role in motor coordination and balance [5]. Neuronal damage in cerebellar stroke arises from oxygen-glucose deprivation (OGD), which triggers complex pathological mechanisms such as ionic dysregulation, altered metabolism, oxidative stress, and inflammation [21, 22]. Purkinje cells, essential for cerebellar function, are highly susceptible to hypoxic injury, leading to severe and often irreversible consequences for stroke survivors [5, 23]. Current stroke therapies, such as thrombolysis or thrombectomy, primarily focus on restoring blood flow and preventing recurrence [24], with few options that directly target the molecular cascades underlying neuronal injury. Thus, developing cell protective strategies that mitigate cell death could significantly improve patient outcomes.

In this study, we investigated the potential cell protective effects of Edaravone, a radical scavenger known for its efficacy in mitigating oxidative stress, particularly in the context of stroke [10, 25, 26]. Using an OGD model in organotypic slice cultures from rat cerebellum and hippocampus, we simulated ischemic stroke conditions and evaluated Edaravone’s ability to reduce cellular damage [19]. The hippocampus, a region highly sensitive to hypoxia [27], was used as a comparative structure in this study, as prior studies have demonstrated Edaravone’s neuroprotective effects specifically in hippocampal tissue [28–30]. This provided a robust benchmark to assess the strength of Edaravone’s protective effects in the cerebellum.

Our findings confirm that OGD effectively induces hypoxia, as evidenced by significant upregulation of HIF-1α and HIF-2α mRNA in both cerebellar and hippocampal tissues [31]. These markers are crucial indicators of hypoxic response [32]. Interestingly, while both tissues exhibited elevated HIF mRNA levels, the protein analysis revealed tissue-specific differences. In the cerebellum, HIF-2α protein levels were significantly increased, whereas HIF-1α was significantly upregulated in the hippocampus. These discrepancies could be attributed to the cellular composition of the tissues. HIF-1α is predominantly expressed in neurons, whereas HIF-2α is primarily found in non-neuronal cells such as endothelial cells and astrocytes [33]. Especially endothelial cells play a central role in the expression of HIF-2α under hypoxic conditions [34]. Given the cerebellum’s relatively higher density of endothelial cells, the stronger HIF-2α response likely reflects the endothelial contribution to hypoxic adaptation in this tissue [35]. The lack of significant HIF-1α upregulation in the cerebellum at the 4-hour time point might be explained by differences in mechanisms of proteostasis or the metabolic activity of cerebellar neurons compared to hippocampal neurons. This difference could be attributed to the fact that the hippocampus is a brain region that shows a particularly strong accumulation of HIF-1α under hypoxia [36]. Additionally, the higher LDH levels observed in cerebellar cultures after OGD suggest more pronounced cellular damage compared to the hippocampus, which could significantly influence the observed differences in HIF expression. The selective loss of cerebellar granule cells, as demonstrated in our previous work [19], may have led to a reduced contribution of HIF-1α-producing neuronal populations, whereas the remaining non-neuronal cells, such as astrocytes and endothelial cells, could continue to upregulate HIF-2α. The greater extent of cell damage in the cerebellum could thus contribute to the differential expression patterns observed. While cerebellar neurons might degrade HIF-1α protein more rapidly during reoxygenation, limiting its accumulation, the cerebellum’s hypoxic response might be dominated by endothelial pathways, reducing the relative contribution of neuronal HIF-1α. These tissue-specific dynamics highlight the complexity of the cerebellum’s response to hypoxia and suggest that its vulnerability might not solely depend on neuronal factors. Further studies focusing on cell-type-specific dynamics of HIF expression and stability are needed to clarify these mechanisms and their implications for therapeutic interventions like Edaravone.

The selection of the 4 h OGD time point for Edaravone treatment was based on our observation that hypoxia-induced cell responses were well-established at this stage. Although earlier cellular responses were detectable after 1 h, longer OGD durations likely provide a more clinically relevant scenario, as stroke patients typically present after a delay [37, 38]. This approach aligns with therapeutic windows for lysis or thrombectomy, making Edaravone a potential adjunct treatment during revascularization and reoxygenation [24]. Future studies should explore the timing and efficacy of Edaravone administration, including pre-hypoxia applications, particularly in high-risk stroke models or surgical scenarios where ischemic events are anticipated. Considering its potent antioxidant properties and established safety profile, Edaravone has the potential to serve as a prophylactic agent, preconditioning neuronal tissue against ischemic injury, mitigating oxidative damage, and reducing the impact of anticipated hypoxic conditions [39].

While previous studies have already indicated the neuroprotective effect of Edaravone in hypoxic cell damage [40], in our study Edaravone also demonstrated significant protective effects in both cerebellar and hippocampal tissues, as indicated by reduced LDH levels in OGD-treated samples. LDH release, a marker of cellular damage, is triggered by membrane permeability loss during hypoxia [41]. Edaravone significantly reduced LDH levels in both tissues, indicating comparable cellular protection in cerebellar and hippocampal tissues, a finding consistent with previous studies on hippocampal protection [28, 30]. However, we observed that the cerebellum experienced a higher percentage increase in LDH levels compared to the hippocampus under OGD conditions. This could suggest greater vulnerability to hypoxia in the cerebellum, though the hippocampus is known to be highly sensitive to hypoxic damage [42]. While the cerebellum showed more pronounced cellular damage, the hippocampus may suffer more from functional impairments due to its involvement in cognitive functions such as memory and learning [43]. This heightened susceptibility is attributed to the vulnerability of hippocampal pyramidal cells and fast-spiking interneurons, which are essential for hippocampal function [44, 45]. Their loss leads to significant functional deficits [43]. Since our assay focused on cellular damage (LDH release), rather than functional deficits, we may not fully capture the hippocampal vulnerability. Future studies incorporating functional assessments would provide a clearer picture of Edaravone’s protective effects.

In addition to its effects on cellular damage, Edaravone significantly reduced ROS levels in both cerebellar and hippocampal slice cultures, emphasizing its role as an effective radical scavenger [10]. OGD conditions induced an increase in ROS production, which persisted through the reoxygenation phase, further exacerbating cell damage. Edaravone’s ability to mitigate this oxidative burst highlights its therapeutic potential in limiting reperfusion injury, a major contributor to neuronal death following stroke [9, 46]. Notably, cerebellar cultures showed higher ROS levels than hippocampal cultures after 4 h OGD, which might reflect the cerebellum’s distinct cellular composition and metabolic demands. Previous studies have shown that the cerebellum, along with the brain stem, exhibits the highest basal rate of ROS production [47]. The relatively high density of astrocytes in the hippocampus, which are known for their antioxidative metabolism and increased glutathione synthesis, may contribute to lower ROS levels under hypoxic conditions [48]. In contrast, Purkinje cells, with their extensive dendritic arborization and elevated metabolic activity, may further amplify ROS production in the cerebellum [49]. These factors could explain the cerebellum’s heightened oxidative response and its relatively higher ROS levels during hypoxia. Our findings indicate that Edaravone’s radical-scavenging effects were more pronounced in the cerebellum, potentially due to the higher initial ROS levels, which provide a greater window for antioxidative intervention. However, excessive ROS levels might also overwhelm Edaravone’s scavenging capacity, partially contributing to less pronounced reduction in LDH levels observed in the cerebellum. It is important to note that elevated ROS levels do not necessarily correlate with greater functional damage. Recent studies have shown that following OGD in hippocampal slice cultures, Edaravone treatment did not attenuate the decline in neuronal transmission [50]. Since our study focused on quantitative cell survival rather than functional assessment, this represents a limitation of our approach and should be further investigated in future studies. The cited study also highlights the importance of superoxide radicals and suggests that Edaravone, as a non-specific radical scavenger, may be less effective compared to other agents specifically targeting super oxides. This aspect requires further exploration to determine the most effective antioxidant strategy.

Mitochondrial dysfunction plays a central role in ischemic neuronal damage, as OGD disrupts oxidative phosphorylation and ATP production [51]. Our findings reveal tissue-specific differences in mitochondrial response to OGD and Edaravone treatment. In cerebellar tissue, Edaravone primarily preserved tissue integrity during reperfusion, as indicated by higher OCR levels after normalization, suggesting that the observed reductions in raw OCR values after OGD were primarily due to cell loss rather than mitochondrial dysfunction. The higher LDH release in cerebellar tissues under OGD conditions reflects greater cellular damage, consistent with the heightened vulnerability of metabolically active Purkinje cells to hypoxic injury [52], which might explain why Edaravone did not influence mitochondrial respiration in cerebellar tissue. In contrast, hippocampal cultures exhibited a distinct mitochondrial response. Both, raw and DNA-normalized OCR data showed a significant enhancement of mitochondrial function in the OGD + Edaravone group which resulted in increased basal respiration and glucose-induced maximal respiration. This suggests that Edaravone not only maintains cellular integrity but also actively enhances mitochondrial activity in this tissue. This may involve mechanisms such as increased metabolic efficiency or mitochondrial biogenesis, potentially mediated by transcription factors like PGC-1α, which regulate mitochondrial adaptation to oxidative stress [53]. The improved mitochondrial function in the hippocampus is further supported by reduced LDH release and lower ROS levels, suggesting a more robust recovery of metabolic and antioxidative capacity. Our findings further support a tissue-specific role of Edaravone in stroke recovery. In cerebellar tissue, the lack of significant changes in DNA-normalized OCR across all conditions suggests that Edaravone’s primary function is cell preservation rather than direct mitochondrial enhancement. The recovery of basal OCR in raw data, despite unchanged DNA-normalized OCR, reinforces this notion, indicating that Edaravone prevents cell loss but does not necessarily improve mitochondrial respiration per cell. In contrast, hippocampal slices exhibited a clear enhancement of mitochondrial function, particularly in glucose-induced OCR, suggesting Edaravone promotes metabolic efficiency post-ischemia. The fact that pyruvate-driven respiration remained unaffected suggests that Edaravone’s effect is primarily linked to glucose metabolism, which represents the primary source of energy for the brain as it fuels the TCA cycle to enhance oxidative phosphorylation. These findings highlight the importance of region-specific metabolic responses in ischemic brain injury and recovery. Edaravone’s effect in the cerebellum appears more structural, whereas in the hippocampus, it enhances cellular metabolism and mitochondrial function, which could have implications for functional recovery and neuroplasticity. These insights emphasize the need for tailored therapeutic approaches depending on the affected brain region, particularly in stroke therapy where metabolic and cellular vulnerabilities differ. The interplay between ROS scavenging and mitochondrial function is particularly evident in the hippocampus. The marked improvement in mitochondrial respiration in hippocampal slices treated with Edaravone suggests that ROS reduction facilitated mitochondrial recovery and may have activated pathways supporting mitochondrial repair or biogenesis. In contrast, the cerebellum’s less efficient mitochondrial recovery, as indicated by basal respiration as well as glucose-induced and pyruvate-induced maximal respiration, could reflect a diminished capacity for adaptive responses under hypoxic and/or OGD stress, potentially due to its distinct cellular composition and metabolic profile [49].

These findings suggest potential tissue-specific roles for Edaravone in stroke therapy. In the cerebellum, Edaravone may primarily help mitigate cell loss and reduce post-stroke atrophy, which could contribute to preserving motor coordination. In the hippocampus, the observed enhancement of mitochondrial function raises the possibility that Edaravone supports cognitive recovery and neuroplasticity. This highlights the broader potential of Edaravone’s neuroprotective effects, which might extend beyond ROS scavenging to include a role in preventing mitochondrial dysfunction. The observed regional disparities further emphasize the importance of tailoring therapeutic strategies to the specific vulnerabilities and recovery capacities of different brain regions. During reperfusion, where oxidative stress and mitochondrial impairment contribute significantly to secondary injury, Edaravone’s ability to mitigate ROS and support mitochondrial recovery highlights its potential as a region-specific therapeutic agent.

Furthermore Edaravone’s neuroprotective effects go beyond ROS scavenging, potentially involving the prevention of brain oedema and modulation of inflammatory pathways [54, 55]. These additional mechanisms could be particularly relevant in cerebellar stroke, where oedema and brainstem compression pose significant risks [56]. While this study focused on cellular damage, future in vivo studies should investigate Edaravone’s broader therapeutic potential, particularly its role in preventing oedema and managing inflammation.

Edaravone might also have potential applications beyond ischemic stroke. For instance, during myocardial infarction or cardiac pump failure, reduced cardiac output leads to cerebral hypoxia, which our OGD model simulates [57]. Edaravone could offer neuroprotection in these contexts, particularly during resuscitation when reperfusion injury may exacerbate neuronal damage. Additionally, its application in perinatal and neonatal asphyxia, where therapeutic hypothermia remains the only treatment for reducing long-term neurological damage, could represent a critical area for future investigation [58].

In summary, our findings indicate that Edaravone provides significant cell protection in both cerebellar and hippocampal tissues under OGD conditions, primarily through its antioxidant properties. These results suggest that Edaravone holds promise as a therapeutic agent for cerebellar stroke, particularly in combination with reperfusion strategies. Future studies should explore Edaravone’s full therapeutic potential in vivo, including its effects on functional recovery, oedema prevention, and broader clinical applications in hypoxic conditions beyond stroke.

## Conclusion

This study highlights the cell protective potential of Edaravone in reducing cellular damage and oxidative stress in cerebellar and hippocampal organotypic slice cultures under OGD-reoxygenation conditions. Our findings reveal tissue-specific responses to ischemic injury and antioxidant treatment, with distinct differences in ROS production, LDH release, and mitochondrial function between the cerebellum and hippocampus. Edaravone’s effectiveness in mitigating these effects highlights its therapeutic value for addressing oxidative stress and ischemic damage. The observed reductions in ROS and LDH levels offer mechanistic insights into its protective action, supporting its application as a post-ischemic intervention. Importantly, our results demonstrate that Edaravone primarily preserves tissue integrity in cerebellar cultures by preventing cell loss rather than directly enhancing mitochondrial function. In contrast, hippocampal cultures exhibited a significant improvement in mitochondrial activity after Edaravone treatment, suggesting additional metabolic benefits such as enhanced mitochondrial efficiency or biogenesis. These findings indicate that Edaravone’s protective effects are not uniform but are dependent on the tissue type, highlighting the need for targeted therapeutic approaches.

In conclusion, Edaravone emerges as a promising therapeutic candidate for cerebellar stroke and other hypoxic brain injuries. Future in vivo studies should investigate its broader therapeutic potential, particularly in conjunction with reperfusion strategies, to prevent neuronal death, preserve tissue integrity, reduce oedema, and enhance long-term neurological recovery.

## Electronic Supplementary Material

Below is the link to the electronic supplementary material.

Supplementary Material 1

## Data Availability

All data supporting the findings of this study are included within the manuscript.

## References

[1] Sarikaya H, Steinlin M. Cerebellar stroke in adults and children. 2018, pp. 301–312. 10.1016/B978-0-444-64189-2.00020-2 29891068

[2] Zhang L, Zhang RL, Jiang Q et al. Focal embolic cerebral ischemia in the rat. *Nature Protocols 2015 10:4* 2015; 10: 539–547. 10.1038/nprot.2015.036 PMC4602402 25741989

[3] Datar S, Rabinstein AA. Cerebellar infarction. Neurol Clin. 2014;32:979–91. 25439292 10.1016/j.ncl.2014.07.007

[4] Ahmed RA, Dmytriw AA, Regenhardt RW, et al. Posterior circulation cerebral infarction: a review of clinical, imaging features, management, and outcomes. Eur J Radiol Open. 2023;11:100523. 37745629 10.1016/j.ejro.2023.100523 PMC10511775

[5] Ioannides K, Tadi P, Lui F et al. Cerebellar Infarct. *StatPearls*, https://www.ncbi.nlm.nih.gov/books/NBK470416/ (2024, accessed 21 October 2024).

[6] Derex L, Cho TH. Mechanical thrombectomy in acute ischemic stroke. Rev Neurol (Paris). 2017;173:106–13. 28238346 10.1016/j.neurol.2016.06.008

[7] Chen YC, Ma NX, Pei ZF, et al. A NeuroD1 AAV-Based Gene Therapy for Functional Brain Repair after Ischemic Injury through in vivo astrocyte-to-Neuron Conversion. Mol Ther. 2020;28:217–34. 31551137 10.1016/j.ymthe.2019.09.003 PMC6952185

[8] Zhao Y, Zhang X, Chen X, et al. Neuronal injuries in cerebral infarction and ischemic stroke: from mechanisms to treatment (review). Int J Mol Med. 2022;49:1–9. 34878154 10.3892/ijmm.2021.5070 PMC8711586

[9] Przykaza Ł. Understanding the Connection Between Common Stroke Comorbidities, Their Associated Inflammation, and the Course of the Cerebral Ischemia/Reperfusion Cascade. *Front Immunol*; 12. Epub ahead of print 15 November 2021. 10.3389/fimmu.2021.782569 10.3389/fimmu.2021.782569 PMC8634336 34868060

[10] Rothstein JD. Edaravone: a new drug approved for ALS. Cell. 2017;171:725. 29100067 10.1016/j.cell.2017.10.011

[11] Takei K, Watanabe K, Yuki S, et al. Edaravone and its clinical development for amyotrophic lateral sclerosis. Amyotroph Lateral Scler Frontotemporal Degener. 2017;18:5–10. 28872907 10.1080/21678421.2017.1353101

[12] Wang G, Su J, Li L et al. Edaravone alleviates hypoxia-acidosis/reoxygenation-induced neuronal injury by activating ERK1/2. *Neurosci Lett*; 543. Epub ahead of print 2013. 10.1016/j.neulet.2013.02.067 10.1016/j.neulet.2013.02.067 23562504

[13] Song Y, Bei Y, Xiao Y, et al., et al. Edaravone, a free radical scavenger, protects neuronal cells’ mitochondria from ischemia by inactivating another new critical factor of the 5-lipoxygenase pathway affecting the arachidonic acid metabolism. Brain Res. 2018;1690. 10.1016/j.brainres.2018.03.006. Epub ahead of print. 10.1016/j.brainres.2018.03.006 29551652

[14] Lee T-H, Uchiyama S, Kusuma Y et al. A systematic-search-and-review of registered pharmacological therapies investigated to improve neuro-recovery after a stroke. Front Neurol; 15. Epub ahead of print 31 January 2024. 10.3389/fneur.2024.1346177 10.3389/fneur.2024.1346177 PMC10866005 38356890

[15] Fu Y, Wang A, Tang R et al. Sublingual Edaravone Dexborneol for the Treatment of Acute Ischemic Stroke. *JAMA Neurol*. Epub ahead of print 19 February 2024. 10.1001/jamaneurol.2023.5716 10.1001/jamaneurol.2023.5716 PMC10877503 38372981

[16] Xu J, Wang A, Meng X, et al. Edaravone Dexborneol Versus Edaravone alone for the treatment of Acute ischemic stroke. Stroke. 2021;52:772–80. 33588596 10.1161/STROKEAHA.120.031197

[17] Zhao K, Li G, Nie L, et al. Edaravone for Acute ischemic stroke: a systematic review and Meta-analysis. Clin Ther. 2022;44:e29–38. 36473732 10.1016/j.clinthera.2022.11.005

[18] Singh S, Kumar A. Protective effect of Edaravone on Cyclophosphamide Induced oxidative stress and neurotoxicity in rats. Curr Drug Saf. 2019;14:209. 31057112 10.2174/1574886314666190506100717 PMC6864589

[19] Wolters A, Reuther J, Gude P, et al. Teriflunomide provides protective properties after oxygen-glucose-deprivation in hippocampal and cerebellar slice cultures. Neural Regen Res. 2021;16:2243–9. 33818508 10.4103/1673-5374.310689 PMC8354112

[20] Althaus O, ter Jung N, Stahlke S, et al. Region-specific protective effects of monomethyl fumarate in cerebellar and hippocampal organotypic slice cultures following oxygen-glucose deprivation. PLoS ONE. 2024;19:e0308635. 39110748 10.1371/journal.pone.0308635 PMC11305562

[21] Weilinger NL, Maslieieva V, Bialecki J et al. Ionotropic receptors and ion channels in ischemic neuronal death and dysfunction. *Acta Pharmacologica Sinica* 2013 34:1 2012; 34: 39–48. 10.1038/aps.2012.95 PMC4086487 22864302

[22] Duris K, Splichal Z, Jurajda M. The role of inflammatory response in Stroke Associated programmed cell death. Curr Neuropharmacol. 2018;16:1365. 29473512 10.2174/1570159X16666180222155833 PMC6251044

[23] Biran V, Heine VM, Verney C, et al. Cerebellar abnormalities following hypoxia alone compared to hypoxic-ischemic forebrain injury in the developing rat brain. Neurobiol Dis. 2011;41:138. 20843479 10.1016/j.nbd.2010.09.001 PMC3910430

[24] Turc G, Bhogal P, Fischer U, et al. European Stroke Organisation (ESO) - European Society for Minimally Invasive Neurological Therapy (ESMINT) guidelines on mechanical thrombectomy in Acute ischemic stroke. J Neurointerv Surg. 2023;15:e8–8. 30808653 10.1136/neurintsurg-2018-014569

[25] Guo L, Pan J, Li F, et al. A novel brain targeted plasma exosomes enhance the neuroprotective efficacy of edaravone in ischemic stroke. IET Nanobiotechnol. 2021;15:107. 34694723 10.1049/nbt2.12003 PMC8675781

[26] Chen C, Li M, Lin L, et al. Clinical effects and safety of edaravone in treatment of acute ischaemic stroke: a meta-analysis of randomized controlled trials. J Clin Pharm Ther. 2021;46:907–17. 33638896 10.1111/jcpt.13392 PMC8359409

[27] Mukandala G, Tynan R, Lanigan S, et al. The effects of Hypoxia and inflammation on synaptic signaling in the CNS. Brain Sci. 2016;6:6. 26901230 10.3390/brainsci6010006 PMC4810176

[28] Ding Y, Zhu W, Kong W, et al. Edaravone attenuates neuronal apoptosis in hippocampus of rat traumatic brain injury model via activation of BDNF/TrkB signaling pathway. Arch Med Sci. 2019;17:514–22. 33747286 10.5114/aoms.2019.89849 PMC7959085

[29] Le S, Zhang P, Li W et al. Pre- and posttreatment with edaravone protects CA1 hippocampus and enhances neurogenesis in the subgranular zone of dentate gyrus after transient global cerebral ischemia in rats. ASN Neuro 6. Epub ahead of print 1 October 2015. 10.1177/1759091414558417/ASSET/IMAGES/LARGE/10.1177_1759091414558417-FIG9.JPEG 10.1177/1759091414558417 PMC4357607 25388889

[30] Wu HT, Yu Y, Li XX, et al. Edaravone attenuates H2O2 or glutamate-induced toxicity in hippocampal neurons and improves AlCl3/D-galactose induced cognitive impairment in mice. Neurotoxicology. 2021;85:68–78. 34004234 10.1016/j.neuro.2021.05.005

[31] Lee JW, Ko J, Ju C et al. Hypoxia signaling in human diseases and therapeutic targets. Experimental & Molecular Medicine 2019 51:6. 2019;51:1–13. 10.1038/s12276-019-0235-1 PMC6586801 31221962

[32] Loboda A, Jozkowicz A, Dulak J. HIF-1 and HIF-2 transcription factors–similar but not identical. Mol Cells. 2010;29:435–42. 20396958 10.1007/s10059-010-0067-2

[33] Barteczek P, Li L, Ernst AS, et al. Neuronal HIF-1α and HIF-2α deficiency improves neuronal survival and sensorimotor function in the early acute phase after ischemic stroke. J Cereb Blood Flow Metabolism. 2017;37:291. 10.1177/0271678X15624933 PMC5363746 26746864

[34] Chen R, Lai UH, Zhu L et al. Reactive oxygen species formation in the brain at different oxygen levels: The role of hypoxia inducible factors. Frontiers in Cell and Developmental Biology 6. Epub ahead of print 10 October 2018. 10.3389/fcell.2018.00132 10.3389/fcell.2018.00132 PMC6192379 30364203

[35] Ventura-Antunes L, Dasgupta OM, Herculano-Houzel S. Resting Rates of Blood Flow and Glucose Use per Neuron Are Proportional to Number of Endothelial Cells Available per Neuron Across Sites in the Rat Brain. Front Integr Neurosci 16. Epub ahead of print 10 June 2022. 10.3389/fnint.2022.821850 10.3389/fnint.2022.821850 PMC9226568 35757100

[36] Berchner-Pfannschmidt U, Frede S, Wotzlaw C, et al. Imaging of the hypoxia-inducible factor pathway: insights into oxygen sensing. Eur Respir J. 2008;32:210–7. 18591338 10.1183/09031936.00013408

[37] Kaneko C, Goto A, Watanabe K, et al. Time to presenting to hospital and associated factors in stroke patients: a hospital-based study in Japan. Swiss Med Wkly. 2011;141. 10.4414/SMW.2011.13296. Epub ahead of print. 10.4414/smw.2011.13296 22161787

[38] Altersberger VL, Wright PR, Schaedelin SA, et al. Effect of admission time on provision of acute stroke treatment at stroke units and stroke centers—An analysis of the Swiss Stroke Registry. Eur Stroke J. 2022;7:117. 35647311 10.1177/23969873221094408 PMC9134779

[39] Sun YY, Li Y, Wali B, et al. Prophylactic edaravone prevents transient hypoxic-ischemic brain Injury: implications for Perioperative Neuroprotection. Stroke. 2015;46:1947–55. 26060244 10.1161/STROKEAHA.115.009162 PMC4480193

[40] Shaki F, Mokhtaran M, Raei M et al. Protective Effects of Edaravone Against Hypoxia-Induced Lethality in Male Swiss Albino Mice. bioRxiv 2021. 2020.05.22.111401.

[41] Coimbra-Costa D, Alva N, Duran M, et al. Oxidative stress and apoptosis after acute respiratory hypoxia and reoxygenation in rat brain. Redox Biol. 2017;12:216. 28259102 10.1016/j.redox.2017.02.014 PMC5334548

[42] Heiss W-D. The ischemic penumbra: correlates in imaging and implications for treatment of ischemic stroke. Cerebrovasc Dis. 2011;32:307–20. 21921593 10.1159/000330462

[43] Grube P, Heuermann C, Rozov A, et al. Transient oxygen-glucose deprivation causes region- and cell type-dependent functional deficits in the mouse Hippocampus in Vitro. eNeuro. 2021;8:221–42. 10.1523/ENEURO.0221-21.2021 PMC8482850 34475264

[44] Einenkel AM, Salameh A. Selective vulnerability of hippocampal CA1 and CA3 pyramidal cells: what are possible pathomechanisms and should more attention be paid to the CA3 region in future studies? J Neurosci Res. 2024;102:e25276. 38284845 10.1002/jnr.25276

[45] Grube P, Heuermann C, Rozov A, et al. Transient oxygen-glucose deprivation causes region- and cell type-dependent functional deficits in the mouse Hippocampus in Vitro. eNeuro. 2021;8:ENEURO0221–212021. 10.1523/ENEURO.0221-21.2021 PMC8482850 34475264

[46] Sun MS, Jin H, Sun X et al. Free Radical Damage in Ischemia-Reperfusion Injury: An Obstacle in Acute Ischemic Stroke after Revascularization Therapy. Oxid Med Cell Longev 2018. Epub ahead of print 2018. 10.1155/2018/3804979 10.1155/2018/3804979 PMC5892600 29770166

[47] Vinokurov AY, Stelmashuk OA, Ukolova PA, et al. Brain region specificity in reactive oxygen species production and maintenance of redox balance. Free Radic Biol Med. 2021;174:195–201. 34400296 10.1016/j.freeradbiomed.2021.08.014

[48] Vilhardt F, Haslund-Vinding J, Jaquet V, et al. Microglia antioxidant systems and redox signalling. Br J Pharmacol. 2016;174:1719. 26754582 10.1111/bph.13426 PMC5446583

[49] Mjaatvedt AE, Wong-Riley MTT. Relationship Between Synaptogenesis and Cytochrome Oxidase Activity in Purkinje Cells of the Developing Rat Cerebellum. 1988. 10.1002/cne.902770202 2852680

[50] Moreton N, Puzio M, O’Connor JJ. The effects of the superoxide dismutase mimetic, MnTMPyP, post hypoxia and oxygen glucose deprivation in isolated rat hippocampal slices. Brain Res Bull. 2022;190:105–15. 36183861 10.1016/j.brainresbull.2022.09.021

[51] Liu F, Lu J, Manaenko A, et al. Mitochondria in ischemic stroke: New Insight and implications. Aging Dis. 2018;9:924–37. 30271667 10.14336/AD.2017.1126 PMC6147588

[52] Au AK, Chen Y, Du L, et al. Ischemia-Induced Autophagy contributes to Neurodegeneration in Cerebellar Purkinje cells in the developing rat brain and in primary cortical neurons in Vitro. Biochim Biophys Acta. 2015;1852:1902. 26071643 10.1016/j.bbadis.2015.06.007 PMC4523442

[53] Rius-Pérez S, Torres-Cuevas I, Millán I, et al. PGC-1 α, inflammation, and oxidative stress: an integrative view in metabolism. Oxid Med Cell Longev. 2020. 10.1155/2020/1452696. Epub ahead of print 2020. 32215168 10.1155/2020/1452696 PMC7085407

[54] Wang G, Su J, Li L, et al. Edaravone alleviates hypoxia-acidosis/reoxygenation-induced neuronal injury by activating ERK1/2. Neurosci Lett. 2013;543:72–7. 23562504 10.1016/j.neulet.2013.02.067

[55] Song Y, Bei Y, Xiao Y, et al. Edaravone, a free radical scavenger, protects neuronal cells’ mitochondria from ischemia by inactivating another new critical factor of the 5-lipoxygenase pathway affecting the arachidonic acid metabolism. Brain Res. 2018;1690:96–104. 29551652 10.1016/j.brainres.2018.03.006

[56] Jensen MB. St. Louis EK. Management of acute cerebellar stroke. Arch Neurol. 2005;62:537–44. 15824250 10.1001/archneur.62.4.537

[57] David H, Ughetto A, Gaudard P, et al. Experimental myocardial infarction elicits time-dependent patterns of vascular hypoxia in Peripheral organs and in the brain. Front Cardiovasc Med. 2021;7:615507. 33585582 10.3389/fcvm.2020.615507 PMC7873295

[58] Rincon F, Mayer SA. Therapeutic hypothermia for brain injury after cardiac arrest. Semin Neurol. 2006;26:387–95. 16969739 10.1055/s-2006-948319

