# Supplementary material for: Differential Protective Effects of Edaravone in Cerebellar and Hippocampal Ischemic Injury Models
Source: Cerebellum. 2025 Feb 18;24(2):49. doi: 10.1007/s12311-025-01804-3 (PMC11835913; doi:10.1007/s12311-025-01804-3)
Supplement: Supplementary file 1 — Supplementary Material 1 [file 12311_2025_1804_MOESM1_ESM.pdf]

Supplementary File 1:

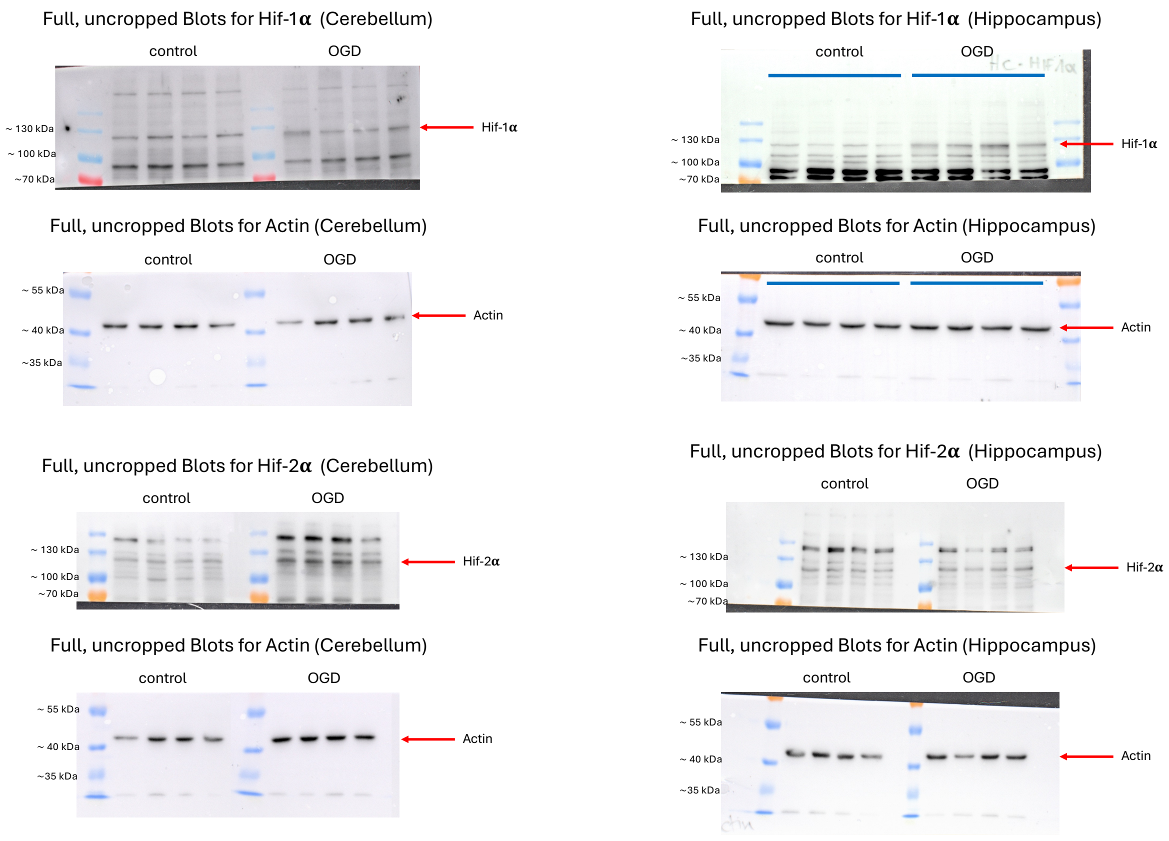

Supplementary Figure 1: Full, uncropped Western blot images for HIF-1α, HIF-2α, and their corresponding actin loading controls. Each blot represents protein expression levels analyzed in cerebellar and hippocampal organotypic slice cultures following OGD conditions. HIF-1α and HIF-2α bands are shown alongside actin to confirm protein loading consistency across samples.
